# A Systematic Review on Carbon Nanotube- and Graphene Nanoplatelet-Reinforced Plasma-Sprayed Alumina-Based Hybrid Nanocomposite Coatings

**DOI:** 10.1021/acsomega.6c02497

**Published:** 2026-05-13

**Authors:** Krishna Kant Pandey, Ashish Ganvir

**Affiliations:** Department of Mechanical and Materials Engineering (MEMA), University of Turku (UTU), Turku 20014, Finland

## Abstract

Carbon nanotubes (CNTs) and graphene nanoplatelets (GNPs) have attracted significant attention as advanced nanomaterial reinforcements for improving the structural and functional performance of ceramic coatings due to their exceptional mechanical strength, thermal stability, and chemical inertness. Among various ceramic matrices, alumina (Al_2_O_3_) remains one of the most widely used coating materials for wear, corrosion, and thermal protection applications. However, its inherent brittleness and susceptibility to crack propagation limit its performance in demanding environments. This review presents a comprehensive and critical analysis of CNT- and GNP-reinforced plasma-sprayed Al_2_O_3_ hybrid nanocomposite coatings. The emphasis has been given on the underlying process–microstructure–property relationships and metallurgical mechanisms governing their performance. The review systematically examines the influence of these reinforcements on particle melting behavior, splat formation, rapid solidification, and interfacial bonding during plasma spraying. Particular attention is given to splat-level phenomena, where CNTs and GNPs play a critical role in improving splat cohesion, reducing intersplat porosity, and inhibiting crack initiation and propagation through crack-bridging and load-transfer mechanisms. The presence of hybrid CNT–GNP reinforcements has been shown to significantly improve coating hardness, fracture toughness, adhesion strength, wear resistance, and corrosion performance compared to monolithic alumina coatings. The synergistic effect of combining one-dimensional CNTs and two-dimensional GNPs enhances reinforcement efficiency by providing multidirectional load transfer and improved stress distribution within the coating microstructure. Furthermore, the review briefly evaluates the effects of reinforcement dispersion techniques, feedstock preparation, and processing routes on the resulting coating microstructure and properties. This review provides fundamental insights into reinforcement mechanisms and microstructure evolution in CNT- and GNP-reinforced ceramic coatings and establishes a framework for the design and optimization of high-performance hybrid nanocomposite coatings for advanced engineering applications.

## Graphical abstract

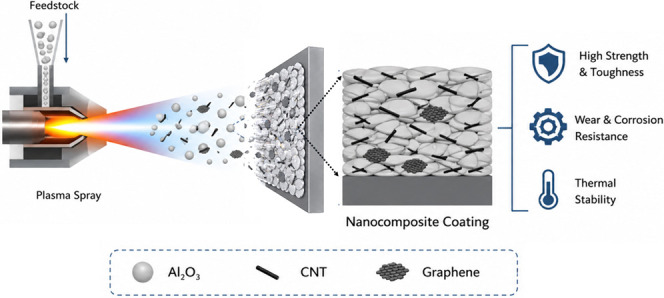

## 1 Introduction

Alumina (Al_2_O_3_) is one of the most extensively used oxide ceramics owing to its outstanding combination of mechanical strength, hardness, thermal stability and chemical inertness. It exhibits a high melting point (∼2050 °C), excellent resistance to wear and remarkable stability in oxidizing and corrosive environments. These attributes make Al_2_O_3_ an indispensable material across a wide range of industrial applications, including electronics, catalysis, biomedical implants, cutting tools and high-temperature structural components.^1–10^ In addition to its bulk uses, Al_2_O_3_ has gained significant importance as a protective coating material for metallic substrates, as shown in Figure 1. When used as a coating, Al_2_O_3_ acts as an effective thermal and chemical barrier, shielding components from oxidation, corrosion and abrasive wear under severe operating conditions. Its electrical insulating nature also makes it suitable for protective layers in electronic and insulating systems. Furthermore, Al_2_O_3_ coatings are widely employed in aerospace, energy, automotive and marine industries where materials are exposed to extreme thermal gradients, mechanical loads, or chemically aggressive environments. The versatility, durability and stability of Al_2_O_3_ continue to drive its demand as a key ceramic for surface protection and functional applications in advanced engineering systems. However, like most monolithic ceramics, Al_2_O_3_ suffers from significant brittleness and poor fracture toughness, which restrict its functionality in structural applications that require mechanical resilience.^8,9,11–14^ This challenge is well-documented in the seminal works of Krell and Schädlich regarding submicrometer grain effects and Parchoviansky et al. on high-temperature behavior, emphasizing the need for secondary phase reinforcements.^8,83,92^ These limitations have driven researchers to develop Al_2_O_3_-based composites by incorporating reinforcing phases, especially at the nanoscale, to overcome inherent brittleness and improve performance.

**Figure 1:**
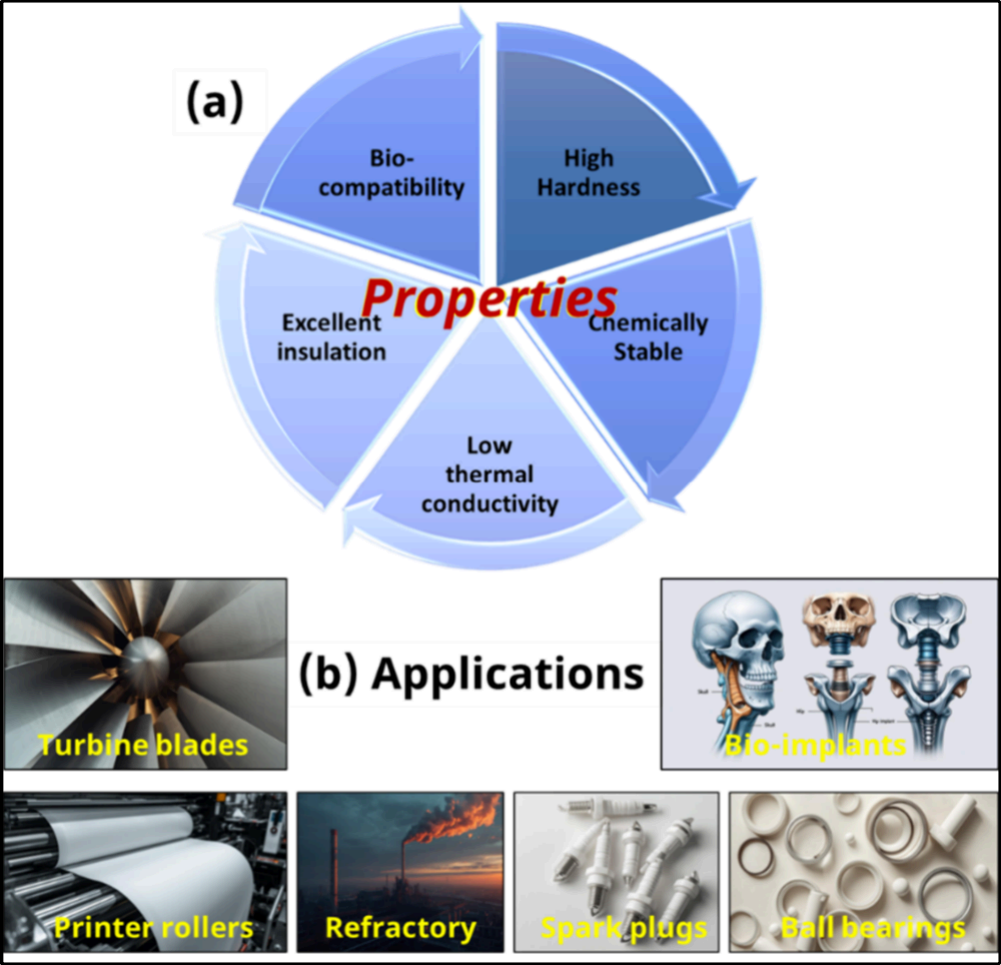
(a) Properties; and (b) applications of Al_2_O_3_ in terms of industrial exposure (Images are partly AI generated).

Among the various reinforcement strategies, the incorporation of carbon-based nanomaterials-specifically carbon nanotubes (CNTs) and graphene nanoplatelets (GNPs)-into ceramic matrices has emerged as a transformative approach. CNTs are one-dimensional tubular structures with extraordinary tensile strength (∼100 GPa), high Young’s modulus (∼1 TPa) and excellent thermal and electrical conductivity. Their hollow cylindrical morphology enables mechanisms like crack bridging, pull-out and fiber stretching, which dissipate energy during fracture and enhance the toughness of brittle ceramics.^15–18^ GNPs, on the other hand, are two-dimensional plate-like structures with high surface area, in-plane strength (∼130 GPa) and superior thermal conductivity (∼5000 W/m·K). GNPs are especially effective in improving barrier properties, refining grain size and facilitating solid lubrication under tribological stress.^19–22^

The addition of CNTs or GNPs individually has demonstrated significant improvements in ceramic composites. Following the trend, recent studies have shown that hybrid reinforcement using both CNTs and GNPs yields synergistic benefits.^23–26^ This is primarily due to the complementary geometries and toughening mechanisms of 1D CNTs and 2D GNPs. The combination allows GNPs to act as bridges between CNT networks, which reduces CNT agglomeration and promotes better dispersion of both phases. Similarly, CNTs can separate stacked GNPs and prevent their restacking, enhancing interfacial contact with the ceramic matrix. This synergy leads to improved microstructural homogeneity, mechanical integrity and multifunctional performance in the resulting composites. Past few decades have shown a remarkable increase in the research dwelling with the hybrid reinforcement of CNTs and GNPs in various metallic, ceramic and polymeric matrices.^23,25–34^ The incorporation of CNTs and GNPs in metallic and polymer matrices markedly enhances both mechanical and functional properties due to their synergistic reinforcement mechanisms. In metallic systems, hybrid CNT-GNP architectures improve strength, hardness and density through efficient load transfer and interfacial bonding. In polymers, their combined 1D–2D network enhances tensile strength, modulus and electrical conductivity while mitigating agglomeration.^19,22,29,34–37^ Together, CNTs and GNPs form interconnected reinforcing networks that optimize stress distribution, crack deflection and conductive pathways. This led to superior multifunctional composites with improved structural and electrical performance. In addition to these metallic and polymeric nanofillers, the inherently brittle nature and poor toughness of Al_2_O_3_ make it an ideal candidate to benefit from CNTs and GNPs hybridization.

To address this issue, Yazdani and group have used this hybrid reinforcement to improve the properties of Al_2_O_3_ nanocomposite via spark plasma sintering (SPS). The nanofillers content was varied from 0 to 1 wt % for GNPs with a fixed 1 wt % CNT content in the Al_2_O_3_ matrix.^33,34,38^ Various tests, such as microhardness, tribology test and electrochemical tests with these various compositions of hybrid matrix were carried out. The results indicated that the amalgamation of 1 wt % CNTs with 0.5 wt % GNPs proved to be the optimum concentration for best performance in all these conditions. For instance, the fracture toughness and flexural strength of this nanocomposite achieved a value of 5.27 MPa·m^1/2^ and 424 MPa, respectively. These values reduced correspondingly by 38 and 35%, after mere increment of GNP content by 0.5 wt %. Hence, it becomes very important to find out an optimum nanofiller content for the best suited performance.

Following the same motivation, Pandey et al. concentrated his works on development of hybrid Al_2_O_3_ matrix reinforced synergistically with 1 wt % CNT and 0.5 wt % GNP. The hybrid nanocomposites were used as a feedstock to fabricate coatings using plasma spraying. Plasma spraying is a versatile and scalable thermal spray technique that allows for the deposition of ceramic coatings onto a wide range of substrates without extensive thermal damage. The basic working principle of a plasma spraying system has been shown in Figure 2. Plasma spraying operates by injecting powder particles into a high-temperature plasma plume where they melt and accelerate toward the substrate, forming “splats” upon impact. The stacking of millions of such splats builds the final coating.^39–46^ However, controlling the microstructure and achieving desired mechanical properties in plasma-sprayed Al_2_O_3_ coatings is challenging. This is mainly due to the inherent issues like splat porosity, weak interlamellar bonding and uneven melting. The introduction of CNTs and GNPs into the plasma spray feedstock addresses many of these challenges. First, the high thermal conductivity of these carbon nanofillers promotes uniform melting and spreading of Al_2_O_3_ particles, resulting in flatter and more continuous splats. Second, the reinforcement phases enhance the interfacial adhesion between splats, reducing delamination and increasing mechanical strength. Third, the nanoscale reinforcements influence the solidification kinetics, grain morphology and porosity of the coating, enabling better control over coating architecture. These effects, collectively, lead to plasma-sprayed coatings with superior wear resistance, toughness and environmental stability.

**Figure 2:**
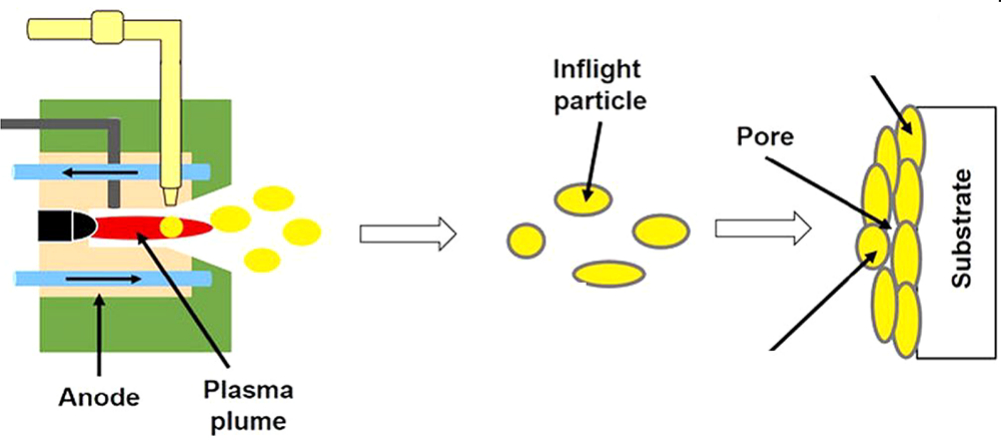
Schematic of atmospheric plasma spraying.^47^ Figure taken/reproduced with permission from Elsevier, copyright [2019].

Despite these advancements, challenges remain in optimizing dispersion techniques, understanding the role of oxidation during spraying, ensuring filler compatibility and minimizing processing-induced defects.^22,35,48–53^ Uniform dispersion of CNTs and GNPs remains a bottleneck due to their strong van der Waals interactions and tendency to agglomerate. This is often addressed using surfactants, ultrasonic agitation, or functionalization techniques, but their effectiveness depends on the specific application. Moreover, thermal degradation of nanofillers during high-temperature spraying must be carefully controlled to preserve their structural integrity.^54–58^ Characterization tools like Raman spectroscopy, SEM/TEM imaging and X-ray diffraction are crucial in validating the presence, phase stability and dispersion quality of the reinforcements.

Despite the growing interest in hybrid reinforcements, a critical gap remains in understanding the splat-level interactions and process-microstructure–property relationships specifically within the rapid solidification environment of plasma spraying. This review aims to provide a comprehensive and critical analysis of CNT- and GNP-reinforced Al_2_O_3_ nanocomposites, with a specific focus on plasma-sprayed coating systems. While previous studies have predominantly explored bulk nanocomposites fabricated through techniques such as spark plasma sintering (SPS) and hot pressing (HP), a systematic understanding of reinforcement behavior under plasma spraying conditions remains limited. In particular, the role of nanocarbon reinforcements in governing splat formation, micromechanical behavior, and interfacial bonding and their subsequent influence on coating-scale and functional performance-has not been comprehensively addressed. This review uniquely bridges this gap by establishing a cross-scale correlation between splat-level phenomena, coating microstructure, and macroscopic properties, including mechanical, tribological, electrochemical, and membrane performance. Furthermore, it provides a critical evaluation of processing strategies, reinforcement dispersion, and structure–property relationships, offering new insights into the synergistic mechanisms of CNT and GNP hybridization. By doing so, this work establishes a unified framework for understanding and optimizing nanocarbon-reinforced alumina coatings for advanced multifunctional applications. It will cover the synthesis techniques, microstructural evolution, splat behavior, mechanical and tribological performance, corrosion resistance, electrical conductivity and membrane applications. The multifunctionality of these hybrid nanocomposites-combining mechanical, electrical, thermal and chemical properties-offers a unique platform for next-generation materials engineering. The review will also present a comparative evaluation of sintering methods like plasma spraying, SPS and hot pressing. Special attention will be given to toughening mechanisms, structure–property relationships and the synergistic roles of CNTs and GNPs. Finally, the review will highlight existing challenges and future research directions to realize the full potential of these advanced ceramic nanocomposites.

## 2 Properties of the Reinforcements

The integration of carbon-based nanostructures such as carbon nanotubes (CNTs) and graphene nanoplatelets (GNPs) into ceramic matrices has revolutionized the design of next-generation ceramic nanocomposites. The schematic representation of CNT and GNP structure has been shown in Figure 3a,b, respectively. While CNTs have a tube-like structure of hexagonally arranged carbon atoms, GNPs exhibit sheet-like arrangement of the carbon atoms. These nanofillers not only enhance mechanical and tribological properties but also provide functionalities such as electrical conductivity, thermal management and corrosion resistance. Their high aspect ratios, nanoscale dimensions and extraordinary intrinsic properties make them ideal reinforcements for traditionally brittle materials like Al_2_O_3_.

**Figure 3:**
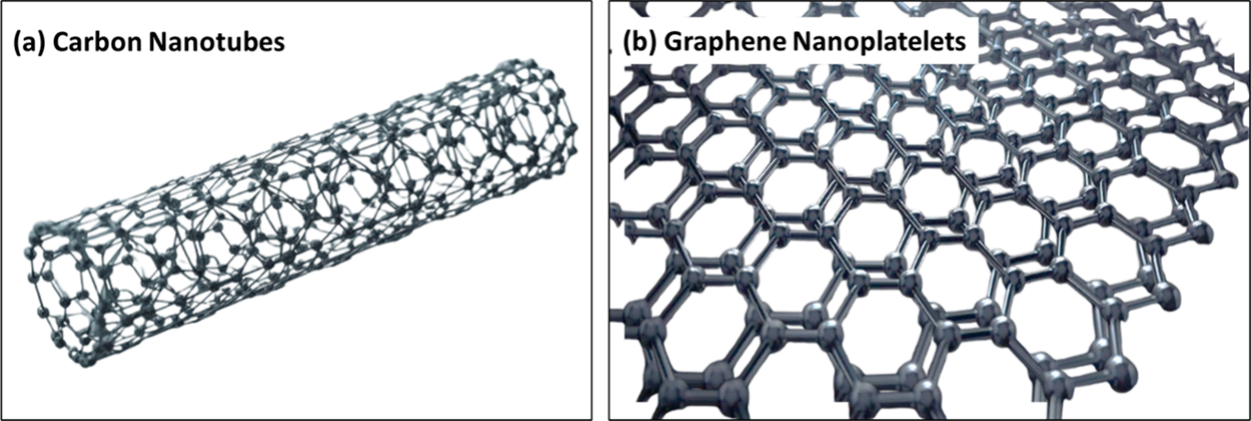
Schematic of (a) CNTs; and (b) GNPs showing the hexagonal arrangement of carbon atoms in tubular and lamellar structure, respectively.

### 2.1 Carbon Nanotubes (CNTs)

Carbon nanotubes (CNTs) are cylindrical nanostructures formed by rolling graphene sheets into seamless tubes and are primarily classified as single-walled (SWCNTs) or multiwalled (MWCNTs) depending on the number of concentric layers. They possess remarkable mechanical properties, including tensile strengths exceeding 100 GPa and a Young’s modulus of about 1 TPa, making them among the strongest known materials.^15,59,60^ Owing to their extremely low density and high aspect ratio (often around 1000), CNTs can establish percolating networks at minimal concentrations, which is advantageous for enhancing mechanical strength, toughness and electrical conductivity in ceramic matrices.

From a reinforcement perspective, CNTs strengthen ceramics through several key mechanisms: (i) crack bridging, where CNTs span microcracks and hinder their propagation; (ii) pull-out, which dissipates fracture energy; (iii) CNT stretching, which absorbs deformation energy; and (iv) bending or kinking, which further delays crack advancement.^61^ These mechanisms collectively improve fracture toughness and mitigate the inherent brittleness of ceramics. The crack-bridging and pull-out mechanisms for CNTs in plasma-sprayed ceramic matrices were fundamentally established by Bakshi et al., who demonstrated that the high-velocity impact of the plasma process requires specific powder treatment to maintain CNT integrity.^62^ Despite these benefits, CNTs face challenges such as agglomeration due to strong van der Waals attractions and structural degradation at elevated processing temperatures. Overcoming these limitations through improved dispersion and controlled processing is crucial for fully exploiting CNTs as efficient reinforcements in advanced ceramic composites.

### 2.2 Graphene Nanoplatelets (GNPs)

Graphene is a two-dimensional (2D) material composed of a single layer of sp^2^-hybridized carbon atoms arranged in a hexagonal lattice. It exhibits exceptional intrinsic properties, including very high in-plane mechanical strength (Young’s modulus ∼ 1 TPa), thermal conductivity exceeding 5000 W/m·K and outstanding electrical conductivity. Graphene nanoplatelets (GNPs) are few-layer stacks of graphene sheets, typically ranging from 1 to 10 μm in lateral size and a few nanometers in thickness, making them practical reinforcements for ceramic composites.^19,21,49,53^ When incorporated into ceramic matrices, GNPs significantly enhance multiple properties. Mechanically, they improve strength and toughness through mechanisms such as grain refinement, load transfer and crack deflection or branching, which hinder crack propagation. Their layered structure also imparts solid lubrication, reducing wear and friction. Similarly, the role of GNPs in creating tortuous crack paths was highlighted by several other works, providing a benchmark for the fracture toughness enhancements observed in hybrid ceramic systems.^53,63,9^ Moreover, GNPs enhance corrosion resistance by forming a dense, impermeable barrier network that limits the penetration of corrosive species. The interconnected graphene network also facilitates thermal and electrical conductivity through percolation pathways. Compared to carbon nanotubes (CNTs), GNPs offer a higher surface area and planar geometry that promote stronger interfacial bonding with the ceramic matrix.^64–66^ However, their tendency to restack due to π–π interactions can diminish these benefits unless effective dispersion and surface modification techniques are applied. Table 1 shows the comparison between different properties of CNTs and GNPs, that make the suitable and favorite candidate for reinforcements.

**Table 1:** Properties Comparison of CNTs and GNPs^20,49,67–73^

property	carbon nanotubes (CNTs)	graphene nanoplatelets (GNPs)
structure	1D tubular (single-walled or multiwalled)	2D sheet-like (few to multilayer graphene)
aspect ratio	very high (length-to-diameter ratio up to 10^3^–10^4^)	moderate (lateral size: 1–25 μm, thickness: few nm)
surface area	∼200–400 m^2^/g (depending on diameter and wall number)	∼500–700 m^2^/g (due to 2D planar structure)
Young’s Modulus	∼1.0 TPa	∼0.8–1.0 TPa
tensile strength	50–150 GPa	80–130 GPa
thermal conductivity	∼2000–3500 W/m·K (along tube axis)	∼3000–5000 W/m·K (in-plane)
electrical conductivity	metallic/semimetallic, up to ∼10^6^ S/m	∼10^5^–10^6^ S/m (depending on layers and defects)
dispersion in ceramics	prone to agglomeration due to van der Waals forces; difficult to disperse homogeneously	better dispersion compared to CNTs, though stacking of platelets may occur
main limitation	difficult dispersion; oxidation at high temperatures	restacking of sheets; may reduce transparency of matrix
synergy in hybrids	provides anchoring and bridging	[provides lubrication and impermeability; complements CNTs

## 3 Synthesis of CNT- and GNP-Reinforced Al_2_O_3_ Nanocomposites

### 3.1 Fabrication of Nanocomposite Powders

For the fabrication of nanocomposite coatings, the nature and characteristics of the feedstock plays a major role. One of the important attested methods used for synthesis of nanocomposite feedstock is spray drying. Spray drying is a versatile method particularly important for ceramic and cermet feedstocks. In this process, fine powders (often in subnanometer level) are first dispersed in a liquid medium to form a slurry. The slurry may include dispersants, binders and solvents to ensure stability and processability.^74–76^ The suspension is then atomized into a drying chamber using a nozzle or rotary atomizer, where droplets rapidly dry in hot air, forming agglomerated spherical granules. The schematic of the process has been shown in Figure 4. Depending on drying conditions and slurry chemistry, the resulting particles can be dense, porous, hollow, or shell-like. A major advantage of spray drying is its ability to produce feedstock with tailored particle sizes and morphologies, including granules made of nanoscale building blocks and was successfully used to synthesize CNTs and GNPs reinforced Al_2_O_3_ nanocomposite powders by pandey and co-workers.^26,75,77,78^ Nonetheless, spray drying remains one of the most flexible and widely applied methods for thermal spray powder preparation.

**Figure 4:**
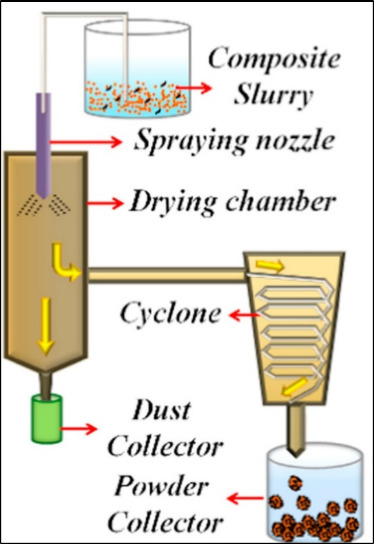
Schematic representation of spray drying process.^78^ Figure taken/reproduced with permission from Elsevier, copyright [2017].

Spray drying stands out among thermal spray powder preparation techniques because of its versatility, compositional control and scalability. Unlike atomization, which is limited mainly to metals and alloys, spray drying can be applied to ceramics, cermets and composite systems, making it particularly suitable for thermal barrier coatings, wear-resistant ceramics and hybrid nanocomposites. By starting from a slurry, spray drying allows the incorporation of nanoscale particles, reinforcements such as CNTs or GNPs, or multiple oxide phases into a single agglomerated granule—something that fused and crushed or milled and sintered powders cannot easily achieve.^74–77^ Another key advantage lies in the morphology of the powders. Spray-dried powders are generally spherical or rounded granules, offering superior flowability compared to angular fused and crushed powders. This ensures consistent powder feeding into the plasma torch or HVOF gun, which directly translates to more uniform coatings. Furthermore, spray drying permits control over granule density and internal structure. For instance, porous or hollow granules can be engineered to improve melting during spraying, whereas dense granules can be optimized for high deposition efficiency.

### 3.2 Fabrication of Nanocomposite Bulk and Coatings

The bulk synthesis technique plays a decisive role in determining the final microstructure, dispersion of reinforcements and multifunctional performance of CNT- and GNP-reinforced Al_2_O_3_ nanocomposites. Among the most relevant processing approaches are plasma spraying, spark plasma sintering (SPS) and hot pressing (HP). Each method affects filler distribution, phase composition, grain size and porosity differently, which in turn influences mechanical, electrical, tribological and corrosion resistance properties.

#### 3.2.1 Plasma Spraying

Plasma spraying is the most widely employed technique for developing coatings and membranes in real-world applications.^79–82^ Plasma spraying involves the injection of powder particles into a high-temperature plasma jet generated by ionizing an inert gas such as argon, nitrogen, or hydrogen. The plasma, with temperatures exceeding 10,000 K, melts or partially melts the powders, which are then propelled at high velocity toward a prepared substrate. Upon impact, the molten droplets flatten into splats, rapidly solidify and build up layer by layer to form a coating. The rapid solidification of these splats builds the lamellar microstructure characteristic of plasma-sprayed coatings. The process allows deposition of ceramics, metals and composites on diverse substrates, offering controlled microstructures, high deposition rates and strong adhesion suitable for protective and functional coatings. The incorporation of CNTs and GNPs into plasma-sprayed Al_2_O_3_ coatings has been shown to refine splat morphology, enhance adhesion and reduce porosity.^23,83–85^ For example, Pandey and co-workers demonstrated that hybrid CNT+GNP reinforced Al_2_O_3_ splats exhibit significantly higher hardness (up to 51 GPa) and elastic modulus (270 GPa) compared to pure Al_2_O_3_ splats, with interfacial adhesion strengths nearly five times higher, as revealed through nanoindentation and nanoscratch tests.^24^ These improvements were attributed to the high thermal conductivity of the nanofillers, which promotes uniform melting and better spreading of molten Al_2_O_3_. Extending the functionality further, plasma-sprayed nanocomposite membranes demonstrated enhanced dye rejection (98.5%) and flux recovery (97.3%), showing the potential of this technique for environmental filtration.^86^ Thus, plasma spraying offers scalability, versatility and multifunctionality, making it the most promising route for surface engineering applications.

#### 3.2.2 Spark Plasma Sintering

In contrast, spark Plasma Sintering (SPS) is a rapid powder consolidation technique that applies uniaxial pressure and pulsed direct current simultaneously to a powder compact within a conductive die. The pulsed current induces localized joule heating and plasma discharges between particles, promoting surface diffusion, neck formation and densification at lower temperatures and shorter times than conventional sintering. The high heating rates (up to 1000 °C/min) minimize grain growth, allowing near-full densification while retaining fine microstructures. SPS is particularly effective for nanocomposites, ceramics and difficult-to-sinter materials, offering superior control over density, grain size and phase composition. Yazdani et al. compared SPS and HP processing for Al_2_O_3_ reinforced with hybrid GNPs and CNTs.^38^ They reported that SPS samples, though densified rapidly, showed larger grain sizes and mixed inter/transgranular fracture modes compared to HP samples. The mechanical property peaks for SPS occurred at relatively lower reinforcement contents, suggesting that rapid heating forced fillers into grain boundaries, limiting their toughening effectiveness. Nevertheless, SPS still achieved flexural strengths >400 MPa and fracture toughness values of ∼5.5 MPa·m^1/2^, demonstrating its effectiveness. In another work, Yazdani et al. highlighted the superior tribological performance of SPS-prepared GNP/CNT hybrid nanocomposites, confirming the synergy of hybrid reinforcement in bulk systems.^33,34,38^ The SPS process, however, is restricted to small sample sizes and requires expensive equipment, which limits its scalability compared to plasma spraying. While Yazdani et al. (38) compared SPS and HP, the broader effectiveness of SPS in retaining the structural integrity of carbon nanostructures was extensively explored by Porwal et al. and Nieto et al., who demonstrated the importance of rapid consolidation in preventing the oxidation of GNPs.^20,87^

#### 3.2.3 Hot Pressing

Hot pressing consolidates powders into dense solids by applying simultaneous high temperature and uniaxial pressure within a die. The combination of heat and pressure enhances atomic diffusion and plastic deformation, accelerating densification and reducing porosity. Typically conducted in vacuum or inert atmospheres, the process achieves high density and mechanical strength, especially for ceramics and composites. Compared to conventional sintering, hot pressing yields finer microstructures and improved mechanical properties but is limited to simple geometries due to die constraints. It remains a reliable technique for producing dense, high-performance materials with excellent structural integrity. A comparative study revealed by Yazdani and co-workers that HP-produced Al_2_O_3_–GNP/CNT nanocomposites exhibited finer grain structures, more uniform filler distribution and superior fracture toughness compared to SPS samples processed under identical nominal conditions.^38^ This was attributed to reduced heating rates, which prevented exaggerated grain growth and promoted better interfacial bonding between Al_2_O_3_ grains and nanofillers.^88^

Table 2 represents a comparative analysis of these three major processing techniques—namely Plasma Spraying, Spark Plasma Sintering (SPS), and Hot Pressing (HP)—in terms of their processing conditions, microstructural control, scalability, and practical considerations. It also shows how processing influences microstructure and reinforcement effectiveness.

**Table 2:** Comparison between Plasma Spraying, Spark Plasma Sintering (SPS), and Hot Pressing

parameter	plasma spraying	SPS	hot pressing
temperature	>10,000 °C (plasma jet)	1200–1650 °C	1600–1700 °C
filler retention	high (with optimized control)	moderate (possible degradation)	high
grain size control	moderate	excellent	good
porosity	∼5–15% (can be controlled)	<2%	<2%
sample geometry	Coatings on complex surfaces	small bulk discs	small to medium-size billets
scalability	high	low	medium
equipment cost	medium	high	medium
references	^79–81,89–91^	^87,92–95^	^88,88^

When these methods are compared, their distinctions become clear. Plasma spraying is uniquely advantageous for coatings, enabling high-throughput, large-scale deposition on complex substrates, but requires careful optimization to preserve filler properties. SPS offers unparalleled control over densification and microstructure refinement at the laboratory scale but struggles with scalability. Hot pressing provides a balance of density and property control but is slower and geometry-limited. Thus, the choice of synthesis technique must align with the desired application: plasma spraying for multifunctional coatings, SPS for small bulk parts with superior nanoscale tailoring and HP for robust medium-sized components with controlled microstructures. A further layer of optimization involves feedstock engineering—spray-drying, surfactant-assisted ultrasonication and functionalization of CNTs/GNPs—to ensure homogeneity, minimize agglomeration and improve filler–matrix bonding, regardless of the processing method. Among the available fabrication techniques, plasma spraying offers unique advantages for coating applications, while SPS and HP are suitable for dense bulk parts. The interplay between filler morphology, dispersion, interfacial bonding and processing route ultimately governs the performance of these advanced nanocomposites. A comparative evaluation of these techniques shows that while SPS excels in grain size control and fast processing, plasma spraying remains unmatched in scalability and application flexibility. The choice of synthesis method, therefore, depends on the target application—whether it be wear-resistant coatings, electrically conductive layers, or dense structural parts.

## 4 Plasma-Sprayed CNT- and GNP-Reinforced Nanocomposites

### 4.1 Splat Behavior in CNT- and GNP-Reinforced Plasma-Sprayed Al_2_O_3_

The study of splats, the fundamental building blocks of plasma-sprayed coatings, provides unique insights into how nanoscale reinforcements influence microstructure formation during deposition. Pandey et al. carried out an extensive work on the development of different kind of splats of CNT and GNP reinforced Al_2_O_3_ nanocomposites and studied the effect of these reinforcements on the mechanical and adhesion properties of the splats.^24,96^ In conventional plasma-sprayed Al_2_O_3_, splats are often irregular in shape, with a high proportion of splash-type morphologies characterized by lobes, cracks and incomplete spreading. These features arise from the high melting point of Al_2_O_3_, its poor wetting characteristics and the extremely rapid solidification rates typical of plasma spray processes, as shown in Figure 5. The incorporation of carbon-based nanostructures, specifically carbon nanotubes (CNTs) and graphene nanoplatelets (GNPs), significantly modifies splat formation by influencing droplet melting, spreading kinetics and solidification dynamics.

**Figure 5:**
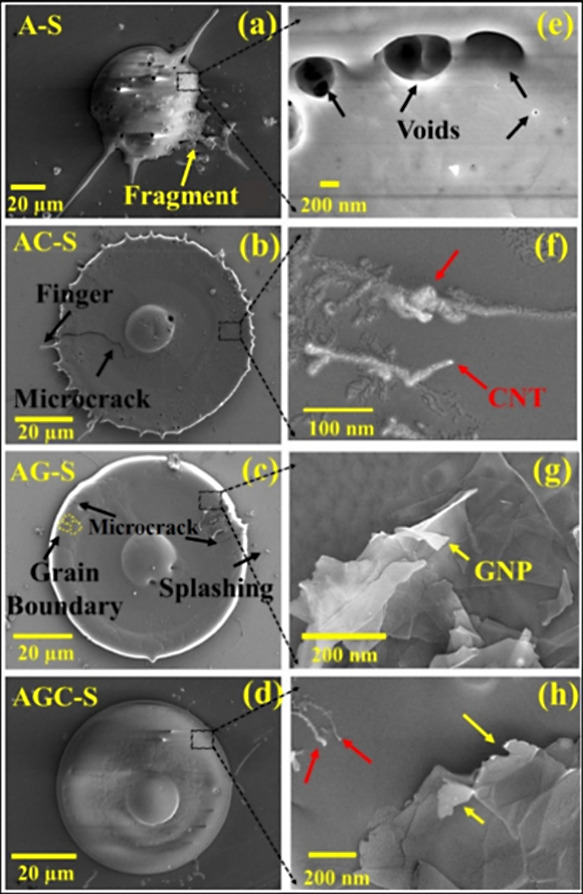
(a–d) Dependence of splat structure with the CNTs and GNPs reinforcement in Al_2_O_3_ nanocomposite; and (e–h) their corresponding high magnification images showing the embedded nanofillers in the matrix.^96^ Figure taken/reproduced with permission from Elsevier, copyright [2019].

A systematic investigation using in situ nanoindentation and high-resolution microscopy revealed that the addition of CNTs and GNPs transformed the splat morphology from irregular forms to more uniform disk-shaped lamellae. The presence of GNPs facilitated rapid lateral heat transfer during droplet impact, minimizing premature edge solidification and enabling complete radial spreading. CNTs, due to their fibrous geometry, became embedded within splats and contributed to improved anchoring on the substrate surface. When used in combination, CNTs and GNPs exhibited a synergistic effect, with each mitigating the other’s tendency to agglomerate, resulting in a higher fraction of sufficiently flattened splats compared to single-filler systems. Quantitative analyses showed a marked increase in the proportion of disk-shaped splats, rising from less than half in pure Al_2_O_3_ to more than three-quarters in hybrid CNT–GNP reinforced systems. It is speculated that the uniform disc shaped splats have the ability to yield not only dense microstructure, but a strong adhesion with the substrates too.^39–40,42^

In addition to morphological changes, the incorporation of CNTs and GNPs influenced splat-level defects such as cracks and interfacial gaps, as shown in Figure 8. Pure Al_2_O_3_ splats frequently exhibited edge cracking due to thermal stresses, while reinforced splats displayed smoother peripheries and fewer quench-induced defects. Microscopic analysis revealed that GNPs were preferentially located at splat boundaries, where they acted as planar bridges that mitigated intersplat separation. CNTs, conversely, were observed spanning across splat thicknesses, effectively tying the lamella to the substrate or underlying splats. Such distribution patterns suggested an active role of reinforcements in modifying splat solidification and adhesion. These microstructural modifications were corroborated by Asiq et al., who emphasized the critical role of uniform filler dispersion and interfacial interactions in nanocomposite formation during high-temperature processing.^97^

At the splat scale, the addition of CNTs and GNPs to plasma-sprayed Al_2_O_3_ fundamentally modifies both electrical and mechanical behavior, with improvements that can be quantified through in situ nanoindentation, scratch testing and electrical measurements. Pure Al_2_O_3_ splats, deposited under conventional plasma conditions, are typically insulating and mechanically brittle, exhibiting hardness values of ∼11–12 GPa and elastic modulus in the range of 200–220 GPa. Various characteristics of the splats in their study has been provided in Table 3. Their load–displacement curves during nanoindentation frequently show discontinuities or “pop-ins,” indicating crack initiation and porosity-driven deformation. The reinforcement of Al_2_O_3_ with CNTs and GNPs significantly improves this response, with hybrid systems consistently outperforming single-filler additions due to synergistic effects. From a mechanical perspective, CNT reinforcement alone increases splat hardness to ∼14–15 GPa, while GNP reinforcement yields ∼15–16 GPa. The improvement arises from better heat conduction during splat spreading (for GNPs) and crack-bridging mechanisms (for CNTs). When CNTs and GNPs are combined, hybrid splats achieve hardness values of ∼18–20 GPa, representing nearly a 60% improvement compared to unreinforced Al_2_O_3_. Similarly, the elastic modulus of pure Al_2_O_3_ splats, measured at ∼220 GPa, rises to ∼240 GPa with CNTs, ∼250 GPa with GNPs and ∼270 GPa with hybrid reinforcements.^24^ These quantitative gains are attributed to reduced porosity, improved splat spreading and phase stabilization of α-Al_2_O_3_, which is harder and denser than the γ-Al_2_O_3_ formed in unreinforced coatings. Mukherjee et al. further demonstrated that hybrid reinforcements enhance fracture toughness by nearly a factor of 2, with energy dissipation mechanisms such as CNT pull-out and GNP crack deflection operating simultaneously.^98^ At the splat level, this translates into fewer cracks around indentation zones and higher resistance to radial crack propagation.

**Table 3:** Microstructural, Mechanical, and Electrical Properties of the Single Splats^24,96^

property		A- splat	AC- splat	AG- splat	ACG- splat	reference
microstructural features		high porosity (10–15%), irregular splats	R=reduced porosity, CNT bridging across splats	improved splat spreading due to thermal conductivity	lowest porosity (5–6%), uniform disk-like splats, synergistic bridging + deflection	^24,96^
splashing fingers	number	12 ± 3	9 ± 2	7 ± 2	3 ± 1	^96^
	length	51 ± 11	13 ± 4.5	7 ± 1.3	2.3 ± 0.8	^96^
hardness (GPa)		11–12	14–15	15–16	18–20	^24^
Elastic Modulus (GPa)		200–220	240	250	270	^24^
adhesion strength (critical load, mN)		5	15	18	25	^24^
conductive area (%)		0 (insulating)	68.30 ± 11.30	78.84 ± 13.67	94.73 ± 6.82	^96^

Adhesion strength of splats is also dramatically improved. Nanoscratch tests show that pure Al_2_O_3_ splats typically delaminate under critical loads of ∼5 mN, whereas CNT- and GNP-reinforced splats withstand ∼ 15–18 mN before detachment. Hybrid CNT/GNP splats resist up to ∼ 25 mN, indicating a nearly 5-fold improvement in adhesion strength compared to unreinforced Al_2_O_3_. This increase is due to CNTs anchoring into substrate asperities during impact and GNPs bridging across intersplat interfaces, thereby sealing microcracks and enhancing cohesion. The improved load transfer between the splat and substrate ensures greater reliability of reinforced coatings under mechanical and thermal stresses. Equally striking improvements are observed in the electrical properties of reinforced splats. Al_2_O_3_ is a classical insulator with resistivity values exceeding 10^9^ Ω·cm. At the splat level, unreinforced Al_2_O_3_ exhibits negligible conductivity (∼10^–9^ S/cm). The incorporation of CNTs or GNPs reduces resistivity by introducing conductive pathways, but the effect is most pronounced when both fillers are used together. The sharp increase is explained by the complementary geometries of CNTs and GNPs: CNTs provide long-range one-dimensional conduction across splat boundaries, while GNPs establish wide-area two-dimensional contacts.^96^ This synergy lowers the percolation threshold and ensures robust conduction pathways across the lamellar structure. Yazdani et al. (2015, *Scientific Reports*) corroborated these findings in SPS-prepared Al_2_O_3_ nanocomposites, reporting similar orders of magnitude increase in conductivity when CNTs and GNPs were combined, even at low loadings.

Mechanistically, the improvements in electrical and mechanical properties of reinforced splats are interconnected. The densification and reduced porosity caused by these reinforcements not only improve hardness and adhesion but also create more continuous networks for charge transport. Similarly, the stabilization of α-Al_2_O_3_, promoted by filler interfaces, enhances hardness while also reducing grain boundary resistivity. Hybrid reinforcements optimize both aspects: CNTs contribute crack bridging and long-range conduction, while GNPs deflect cracks, provide lubricating interfaces and form impermeable barriers against ionic transport.

### 4.2 Plasma-Sprayed CNT/GNP-Reinforced Al_2_O_3_ Nanocomposite Coatings

The incorporation of carbon nanotubes (CNTs) and graphene nanoplatelets (GNPs) into plasma-sprayed Al_2_O_3_ coatings has received significant attention in the past decade as a strategy to overcome the inherent brittleness and microstructural limitations of ceramic coatings. Al_2_O_3_ is widely used for wear protection, corrosion resistance, electrical insulation and thermal barrier applications, yet its plasma-sprayed coatings often suffer from porosity, weak interlamellar cohesion and susceptibility to crack propagation. The development of nanocomposite coatings reinforced with CNTs and GNPs offers a pathway to address these challenges, as the nanofillers modify splat dynamics, improve microstructural cohesion and introduce multifunctional properties.

#### 4.2.1 Microstructural Modifications

The most fundamental effect of CNT and GNP reinforcement in plasma-sprayed Al_2_O_3_ coatings occurs at the splat scale. This change was attributed to enhanced thermal conductivity and better wetting behavior induced by the reinforcements, particularly GNPs, which facilitated lateral heat transfer during impact. CNTs further acted as anchoring agents, embedding into splats and bridging interfaces. These splat-level modifications were corroborated by several researchers, who reported that hybrid CNT–GNP additions in plasma-sprayed Al_2_O_3_ enhanced fracture resistance by crack bridging and deflection at intersplat boundaries.^26,98^

Cross-sectional microscopy of coatings further demonstrated a significant reduction in porosity when these reinforcements were employed, as shown in Figure 6a. Pure Al_2_O_3_ coatings typically exhibited porosity levels exceeding 10%, much of it interconnected, whereas hybrid CNT/GNP coatings achieved porosities closer to 5–6%, with the pores being isolated and closed. Such microstructural refinement was attributed not only to improved splat spreading but also to the grain boundary pinning effect of the nanofillers, which inhibited abnormal grain growth during rapid quenching. Similar densification effects were reported by Asiq et al. in spark plasma sintered Al_2_O_3_ nanocomposites, where uniform dispersion of nanofillers led to improved grain refinement and load transfer.^97^ Thus, although processing methods differ, the fundamental role of CNTs and GNPs in refining Al_2_O_3_ microstructures appears universal.

**Figure 6:**
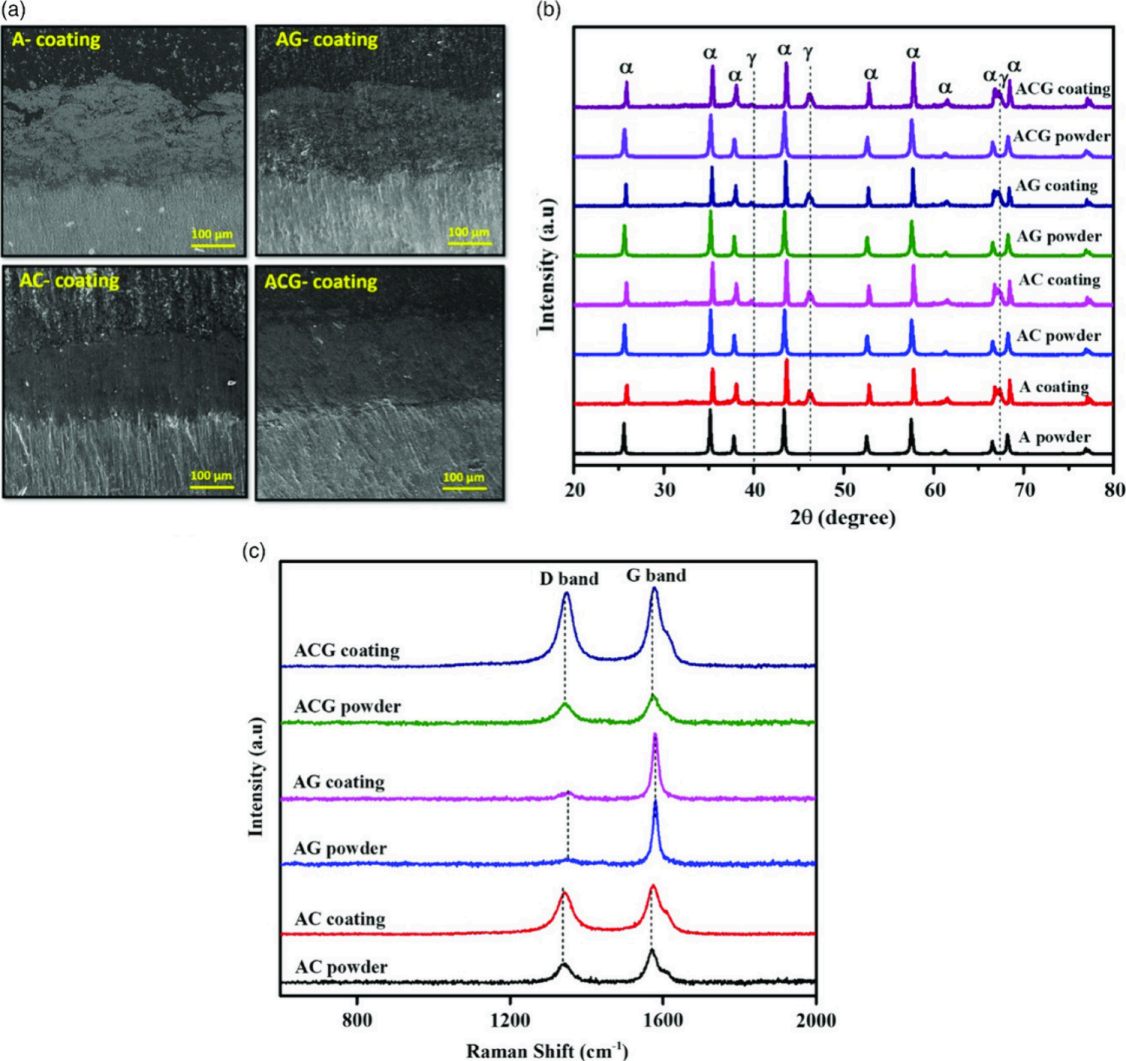
(a) Cross-sectional imaging of Al_2_O_3_ coatings showing the effect of reinforcement on the coating densification; (b) XRD of the powders and Al_2_O_3_ and reinforced powders and the corresponding coatings; (c) Raman spectra of the coatings, proving the survival of the nanofillers after their exposure in high temperature plasma plume.^26^ Figure taken/reproduced with permission from John Wiley and Sons, copyright [2019].

Figure 6b presents the XRD spectra of the plasma-sprayed coatings in comparison with their corresponding feedstock powders. The as-received powders exhibit a pure corundum structure (α-Al_2_O_3_), whereas the plasma-sprayed coatings show the presence of both α- and γ-Al_2_O_3_ phases. The α-phase can be attributed to unmelted particles or those that experienced slower cooling rates, while the metastable γ-phase forms from molten Al_2_O_3_ droplets that solidified rapidly under high cooling rates during plasma spraying. Notably, no carbide-related peaks were detected at 2θ ≈ 31.5° and 55.0°, confirming the absence of any significant reaction between the carbonaceous reinforcements and Al_2_O_3_ during the process.^98^ However, due to the detection limit of XRD (unable to identify phases below ∼ 5% content), the presence of CNTs and GNPs could not be verified. To confirm their retention, Raman spectroscopy was conducted (Figure 6c). The spectra exhibit distinct D and G bands, confirming the presence of CNTs and GNPs in the plasma-sprayed Al_2_O_3_ matrix. The intensity ratio (I_D_/I_G_) is higher for the coatings compared to the spray-dried powders, indicating increased defect formation resulting from high-temperature and high-impact conditions experienced during spraying. Additionally, a slight shift of the Raman peaks toward higher wavenumbers in the coatings suggests the development of compressive stresses within CNTs and GNPs due to the high-velocity impact of the heated feedstock on the substrate.

#### 4.2.2 Mechanical Properties of CNT/GNP-Reinforced Plasma-Sprayed Al_2_O_3_ Coatings

Before discussing the quantitative improvements in mechanical properties, it is important to understand the fundamental mechanisms through which CNTs and GNPs reinforce the Al_2_O_3_ matrix. The enhancement arises from a combination of complementary nanoscale mechanisms associated with their distinct geometries. CNTs primarily contribute through crack bridging, pull-out, and fiber stretching, which dissipate fracture energy and delay crack propagation. In contrast, GNPs, owing to their two-dimensional planar structure, promote crack deflection, bifurcation, and grain boundary sliding, thereby increasing the crack path and energy required for fracture. Additionally, both reinforcements facilitate effective load transfer from the ceramic matrix due to their high stiffness and aspect ratio. GNPs further introduce a solid lubrication effect, reducing interfacial stresses during deformation. When combined, CNTs and GNPs form an interconnected hybrid network that enables multidirectional stress distribution, suppresses crack initiation, and enhances structural integrity. This synergistic interaction is central to the observed improvements in hardness, fracture toughness, and overall mechanical performance of the nanocomposite coatings.

The mechanical properties of plasma-sprayed Al_2_O_3_ coatings are governed by splat morphology, interlamellar bonding, porosity and phase composition. While pure Al_2_O_3_ coatings are hard, they are also brittle, with low fracture toughness and weak cohesion across splat interfaces. The incorporation of carbon nanotubes (CNTs) and graphene nanoplatelets (GNPs) significantly alters these mechanical characteristics by refining the microstructure, enhancing load transfer across splats and introducing nanoscale toughening mechanisms as proved by Pandey et al. in their works regarding the plasma sprayed splats.^24^ A quantitative evaluation of hardness, elastic modulus, fracture toughness and adhesion reveals that hybrid CNT/GNP reinforcement consistently produces coatings that are mechanically superior to both monolithic Al_2_O_3_ and single-filler composites, as depicted in Figure 7. Bulk microhardness measurements of the hybrid reinforced Al_2_O_3_ coatings echoed these results. Plasma-sprayed coatings reinforced with CNTs and GNPs showed Vickers hardness values in the range of 1100–1300 HV, compared to ∼850–950 HV for pure Al_2_O_3_. The improvements are consistent with the findings of Yazdani et al., who reported that SPS-prepared Al_2_O_3_–GNP nanocomposites exhibited hardness enhancements of ∼25% due to grain refinement and strong interfacial bonding.^38^ Mukherjee and group similarly emphasized the critical role of hybrid fillers in achieving significant hardness gains, linking the effect to a combination of load transfer, crack arrest and microstructural densification.^98^

**Figure 7:**
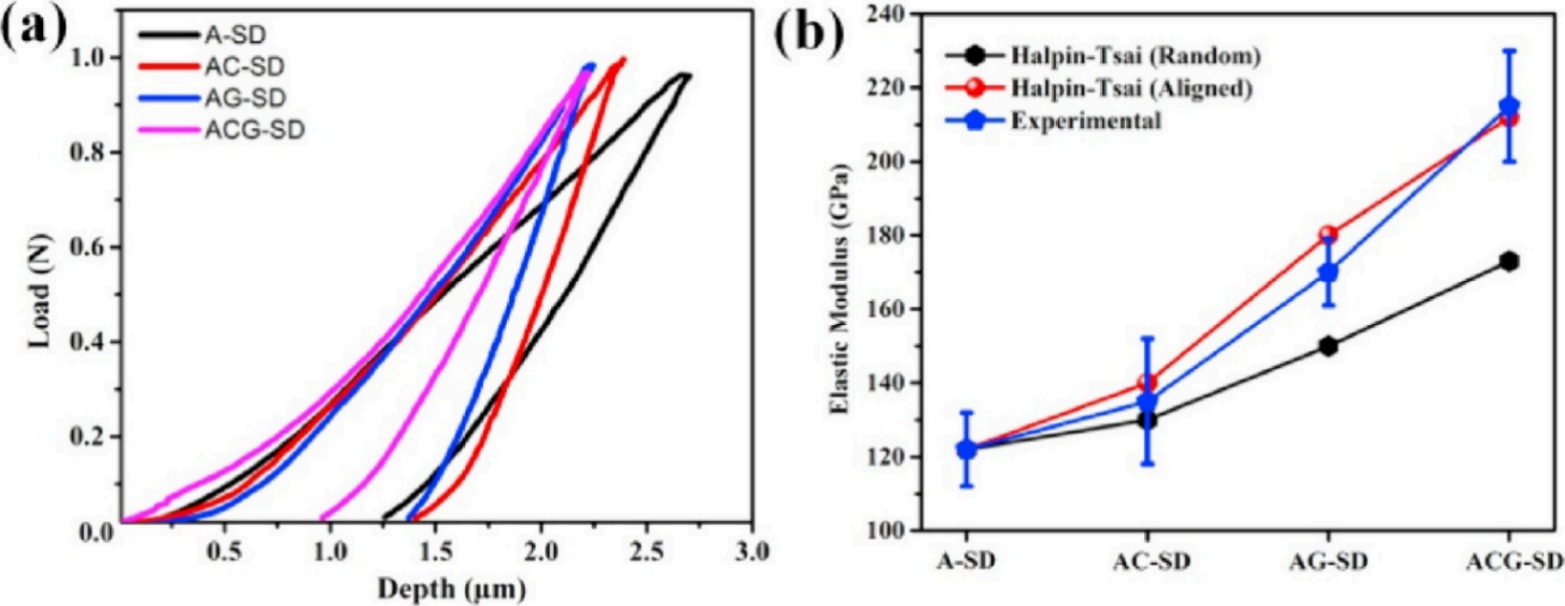
(a) Load vs depth curve obtained from the pico-indentation experiment; (b) line plot quantifying the hardness and elastic modulus values of the different splats.^98^ Figure taken/reproduced with permission from Elsevier, copyright [2017].

Fracture toughness is a limiting factor for the use of monolithic Al_2_O_3_ in mechanical applications, typically reported at ∼3.5–4.0 MPa·m^1/2^ for dense Al_2_O_3_. Plasma-sprayed coatings usually exhibit even lower values due to porosity and interlamellar cracks. The incorporation of CNTs and GNPs markedly enhances fracture toughness through multiple nanoscale toughening mechanisms, as shown in Figure 8. CNTs provide crack bridging, pull-out and elastic stretching, while GNPs deflect cracks, cause branching and increase the crack propagation path length. Mukherjee et al. reported fracture toughness values as high as 5.0–5.7 MPa·m^1/2^ in hybrid CNT/GNP reinforced Al_2_O_3_ prepared via thermal spray methods, representing nearly a 2-fold improvement over monolithic coating.^98^ The reinforced coatings showed resistance to crack initiation and propagation under indentation loads, with reduced pop-ins in load–displacement curves and fewer radial cracks observed around Vickers indents. These results confirm that hybrid reinforcements dissipate fracture energy more effectively, delaying catastrophic crack growth. The group further validated these mechanisms in SPS-prepared composites, emphasizing that the synergy between CNTs and GNPs maximizes interfacial stress transfer and minimizes local stress concentrations.^97,98^ The hardness gains observed in above-mentioned works are consistent with the findings of Centeno et al., who reported that the uniform distribution of graphene sheets creates a ‘lubricating’ grain boundary effect that facilitates grain refinement, a phenomenon also observed in various laser-treated ceramic composites.^9^

**Figure 8:**
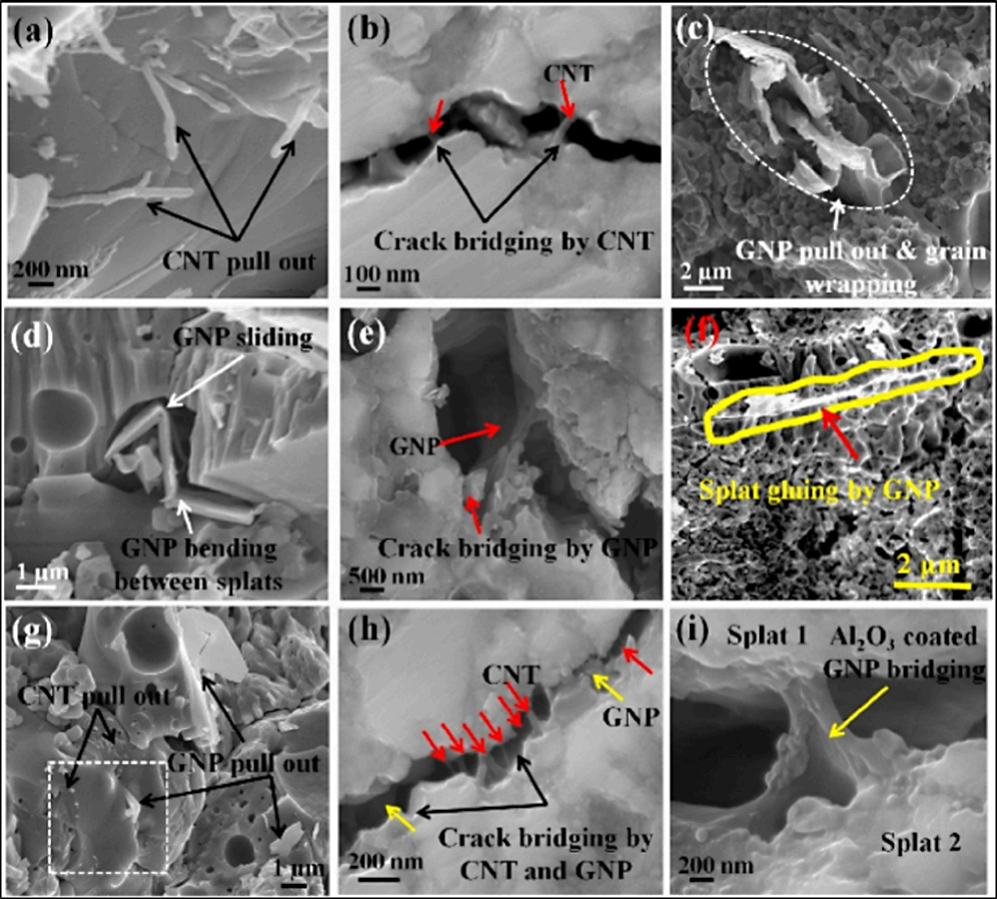
(a–i) Various mechanisms involved in improvement of the mechanical properties of the Al_2_O_3_ coatings after the reinforcement of CNTs and GNPs.^98^ Figure taken/reproduced with permission from Elsevier, copyright [2017].

The remarkable improvements in the mechanical properties of CNT/GNP reinforced plasma-sprayed Al_2_O_3_ coatings can be understood through a combination of microstructural refinement and nanoscale reinforcement mechanisms. One of the most fundamental contributions arises from load transfer. Both CNTs and GNPs possess exceptionally high Young’s moduli (on the order of ∼1 TPa), which are far superior to that of Al_2_O_3_ itself. When these nanofillers are uniformly dispersed within the ceramic matrix, they serve as effective reinforcements, allowing stresses to be redistributed from the relatively weaker Al_2_O_3_ grains to the mechanically stronger carbon nanostructures. This redistribution leads to measurable increases in hardness and modulus, as observed in the splat-level nanoindentation and bulk microhardness results across multiple studies. Another critical contribution is the role of CNTs and GNPs in crack-resistance mechanisms. Al_2_O_3_ coatings are prone to brittle fracture, with cracks readily initiating and propagating along splat boundaries and through grain interfaces.^37,98,29^ CNTs suppress this tendency by bridging cracks and resisting their opening and in many cases, they undergo pull-out and stretching, thereby absorbing fracture energy and delaying catastrophic propagation. GNPs, owing to their two-dimensional platelet structure, interact with cracks differently: they deflect cracks along their basal planes, create branching and force crack paths to become tortuous. This deflection and branching increase the fracture surface area and the energy required for crack progression. When CNTs and GNPs are combined, they work synergistically at different scales, with CNTs controlling microcrack growth and GNPs altering macrocrack trajectories.

#### 4.2.3 Wear Resistance of CNT/GNP-Reinforced Al_2_O_3_ Nanocomposites

Under dry sliding conditions, various researches reported a remarkable reduction in wear and frictional losses in Al_2_O_3_ coatings after the incorporation of CNTs and GNPs. In pristine plasma-sprayed Al_2_O_3_ coatings, the coefficient of friction (CoF) typically ranges from 0.4 to 0.45, owing to the brittle nature of Al_2_O_3_ and the absence of any self-lubricating mechanism. The addition of GNPs alone reduced the CoF to approximately 0.27, primarily due to the formation of graphitic lubrication films on the worn surface during sliding. CNTs contributed further by providing a rolling-lubricant effect, thereby minimizing asperity interactions.^23,25^ When CNTs and GNPs were simultaneously introduced into the Al_2_O_3_ matrix, forming the hybrid ACG (Al_2_O_3_ + CNT + GNP) coating, the synergistic action of one-dimensional CNTs and two-dimensional GNPs resulted in the lowest CoF values, ranging between 0.18 and 0.22.^23,25^ This corresponded to the smoothest wear tracks and minimal material removal. Sony et al. quantified this enhancement by reporting a 93.25% reduction in wear volume loss and a 90.94% decrease in the specific wear rate in the hybrid AGC-SD coating compared to the pristine Al_2_O_3_ coating. Microscopic analysis revealed that unreinforced Al_2_O_3_ coatings suffered from severe wear characterized by grain pull-outs, microcracks and debris accumulation, whereas the hybrid coating exhibited mild wear with the formation of a uniform, continuous transfer film. Similarly, Pandey et al. found that the weight loss in hybrid coatings was reduced by six to seven times compared to pure Al_2_O_3_ coatings. The improved performance was attributed to the formation of a self-lubricating carbon-rich tribofilm at the sliding interface, the enhanced load-bearing capacity due to improved fracture toughness and the lower porosity that minimized stress concentrations. These factors collectively established a smoother sliding regime and increased the coating’s durability under dry wear conditions. A brief illustration of the effect of effect of hybrid carbonaceous nanofillers reinforcement on the wear property of the coatings is represented in Figure 9. It shows the synergistic addition of CNT and GNP enables self-lubrication in the coating during tribology due to simultaneous rolling and sliding effect of these reinforcements.

**Figure 9:**
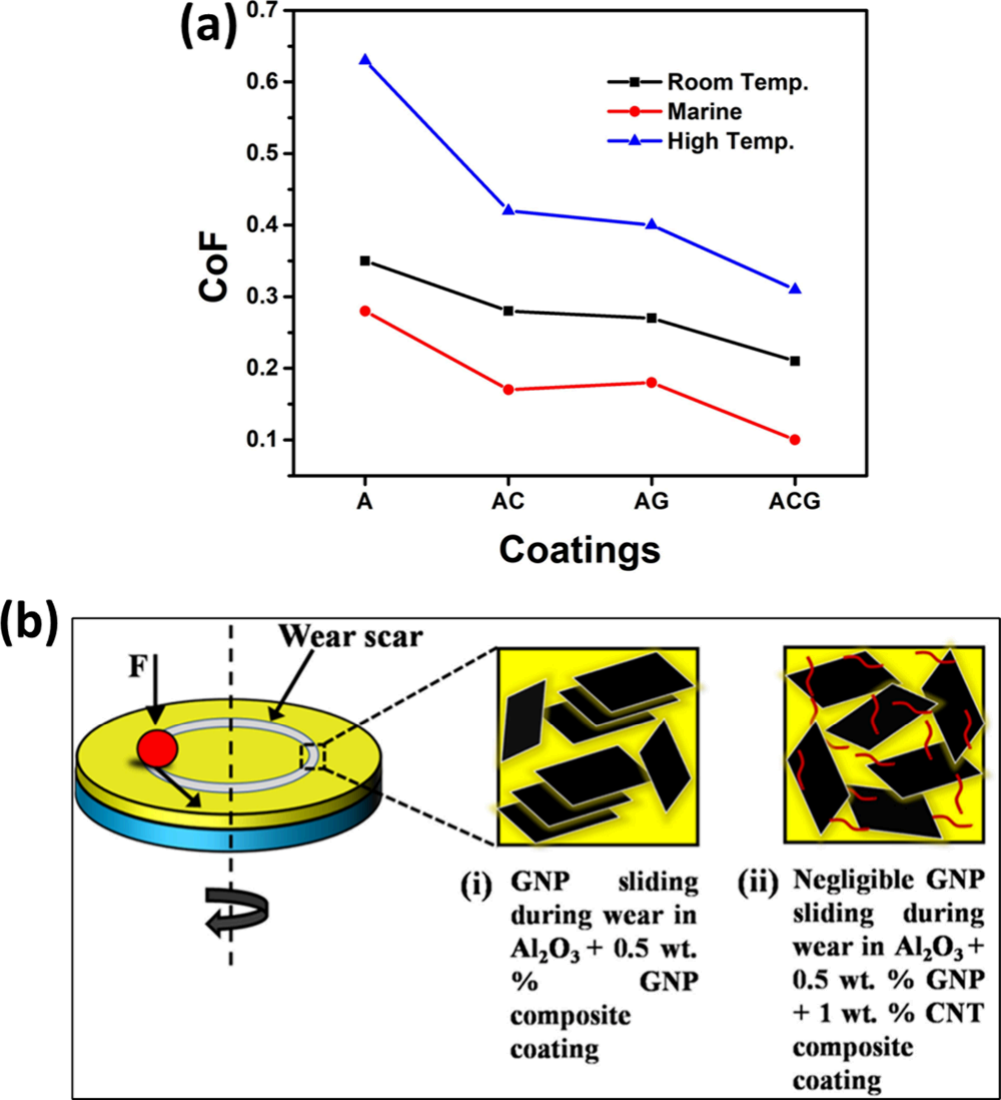
(a) CoF value of different coatings in different sliding conditions; (b) schematic representation of the reduction of COF in AG-SD compared to AGC-SD coatings.^23,25^ Figure taken/reproduced with permission from Elsevier, copyright [2019].

In marine and corrosive environments, where wear is accompanied by ionic corrosion, fluid lubrication and debris entrapment, the inclusion of CNTs and GNPs proved equally beneficial. Pandey et al. investigated the wear performance of such coatings in simulated seawater prepared according to ASTM D1141–98. The study revealed that although all coatings showed slightly lower CoF values due to the lubricating effect of water, the hybrid ACG coating maintained the most stable and lowest CoF in the range of 0.09–0.10, compared to 0.24 for the unreinforced Al_2_O_3_ coating.^23^ In this corrosive medium, CNTs and GNPs not only acted as solid lubricants but also promoted the formation of a stable tribofilm that resisted ionic attack and mechanical erosion. The uniform distribution of the reinforcements provided consistent lubrication, while the enhanced surface smoothness reduced three-body abrasion caused by entrapped wear debris. Energy-dispersive spectroscopy (EDS) mapping of worn tracks further confirmed a homogeneous elemental distribution of aluminum, oxygen, silicon and carbon across the wear surface of the hybrid coating, indicating the development of a continuous protective layer. In contrast, the pure Al_2_O_3_ coating exhibited irregular elemental mapping with significant chlorine accumulation, suggesting susceptibility to corrosion and localized wear. Hence, the hybrid coating provided dual protection—mechanical and chemical—making it suitable for marine and saline environments where conventional ceramic coatings typically fail.

At elevated temperatures around 300 °C, tribological mechanisms undergo transformation due to oxidation, softening of the contact surfaces and potential degradation of lubricants. Even under such demanding conditions, Pandey et al. observed that the hybrid coating demonstrated remarkable stability. Although the absolute CoF values increased for all coatings at high temperatures—from 0.62 for pure Al_2_O_3_ to approximately 0.3 for the hybrid ACG—the relative improvement remained consistent, corresponding to nearly a 50% reduction in friction.^23^ The wear loss, moreover, decreased 6-fold for the hybrid coating compared to the monolithic Al_2_O_3_. Microscopic analysis through scanning electron microscopy (SEM) and optical profilometry revealed significantly narrower wear tracks (200–300 μm for ACG versus more than 500 μm for Al_2_O_3_) and shallower wear depths, reflecting superior resistance to adhesive and oxidative wear. The exceptional high-temperature performance was ascribed to the thermal stability and lubricity of CNTs and GNPs, which continued to form a carbonaceous tribofilm even under heat and stress. Raman spectroscopy further confirmed the structural stability of the graphitic nanofillers; although minor blue shifts in the D and G bands were detected—indicative of lattice compression under thermal and mechanical loads—the overall graphitic nature was preserved. The detection of carbon on worn surfaces validated the persistence of the lubricating film, which mitigated severe oxidation and prevented spallation of the brittle Al_2_O_3_ grains. Thus, even at elevated temperatures, the hybrid coating maintained its low friction and high wear resistance, ensuring reliable performance in thermally demanding applications such as turbines and exhaust systems.

The key factor for the improvement in the wear resistance of the coatings is the formation of a thin, adherent tribofilm at the sliding interface during wear. This carbon-rich film—composed of transferred CNTs, GNPs and oxidized debris—acts as a protective lubricating layer that minimizes direct asperity contact and drastically reduces friction. The tribofilm also functions as a load-bearing intermediary, distributing stress evenly and shielding the brittle ceramic beneath from direct impact.^27,99–102^ Within this system, the two reinforcements contribute distinct yet complementary functions: CNTs act as nanorollers, facilitating smooth rolling motion and reducing shear resistance, while GNPs provide planar shearing and sliding between their stacked layers. Together, they form a dynamic lubricating regime that constantly renews itself through wear debris deposition. Additionally, the toughened microstructure suppresses crack initiation and propagation, while carbonaceous debris between the mating surfaces further stabilizes the sliding process by acting as microrollers. The schematic model proposed by Pandey et al. illustrated this synergy, wherein a thin carbonaceous film adheres to the counterface, transforming the sliding interaction into a controlled, low-friction process that decouples the load from the underlying brittle ceramic.

When comparing single and hybrid reinforcements, it becomes evident that while both enhance tribological performance, the hybrid system offers a superior combination of wear resistance, mechanical stability and environmental adaptability. Sony et al. observed an interesting phenomenon wherein GNP-only coatings exhibited a lower CoF (0.27) than the hybrid coating (0.42) in certain conditions, primarily due to the abundant graphitic lubrication provided by fragmented GNPs.^25^ However, this came at the expense of higher wear and reduced mechanical stability. In contrast, the hybrid coating displayed excellent toughness and lower wear rates, emphasizing that a minor increase in CoF is an acceptable trade-off for significantly enhanced durability. The optimized reinforcement composition—1 wt % CNT combined with 0.5 wt % GNP—was found to provide the best balance between uniform dispersion, coating densification and mechanical strength. Higher concentrations of either filler tended to cause agglomeration and poor cohesion, leading to a decline in toughness and wear resistance. Overall, the synergistic interaction between CNTs and GNPs not only improved lubrication but also reinforced the structural integrity of the Al_2_O_3_ coating, leading to a multifunctional material capable of performing under dry, wet and high-temperature conditions. These results collectively establish hybrid carbon nanofiller reinforcement as a powerful strategy for designing next-generation, wear-resistant ceramic coatings for extreme environments.

#### 4.2.4 Electrochemical Corrosion Resistance of CNT/GNP-Reinforced Al_2_O_3_ Nanocomposites

Al_2_O_3_ has long been used as a protective coating material due to its inherent chemical inertness and ability to withstand harsh environments. However, when processed by plasma spraying, Al_2_O_3_ coatings typically contain significant levels of porosity, microcracks and weak splat interfaces. These structural imperfections create interconnected channels through which electrolytes can penetrate, ultimately reaching the metallic substrate and initiating localized corrosion. As a result, the corrosion protection provided by monolithic plasma-sprayed Al_2_O_3_ is often insufficient for applications in aggressive environments such as marine, aerospace and chemical processing systems. The reinforcement of Al_2_O_3_ coatings with CNTs and GNPs offers an effective strategy to overcome these limitations, improving electrochemical corrosion resistance through microstructural refinement, barrier effects and phase stabilization.

Electrochemical testing provides quantitative evidence of the improvements imparted by CNT/GNP reinforcement. In potentiodynamic polarization studies, pure plasma-sprayed Al_2_O_3_ coatings typically exhibit corrosion current densities (*i*_corr_) in the range of 10^–5^–10^–6^ A/cm^2^, reflecting relatively high corrosion rates due to electrolyte ingress. The outcomes of the electrochemical tests have been provided in Figure 10. The representative plots for the polarization curves of the coatings have been illustrated in Figure 10. Hybrid CNT/GNP reinforced coatings, by contrast, show *i*_corr_ values on the order of 10^–7^ A/cm^2^, nearly an order of magnitude lower. The corrosion potential (*E*_corr_) also shifts positively by 200–300 mV compared to monolithic Al_2_O_3_, indicating enhanced resistance to anodic dissolution. These shifts demonstrate that the reinforcements not only slow down corrosion kinetics but also increase the thermodynamic stability of the coating–substrate system under aggressive electrolytic conditions. Previous researches provided further support by demonstrating that hybrid CNT/GNP reinforcements improve interfacial toughness, thereby reducing the susceptibility of coatings to crack-driven electrochemical degradation. Few researchers observed similar reductions in corrosion activity in SPS-prepared Al_2_O_3_ nanocomposites, suggesting that the electrochemical benefits of CNTs and GNPs reinforcement are independent of processing route and instead arise from fundamental microstructural effects.^33,97,98^

**Figure 10:**
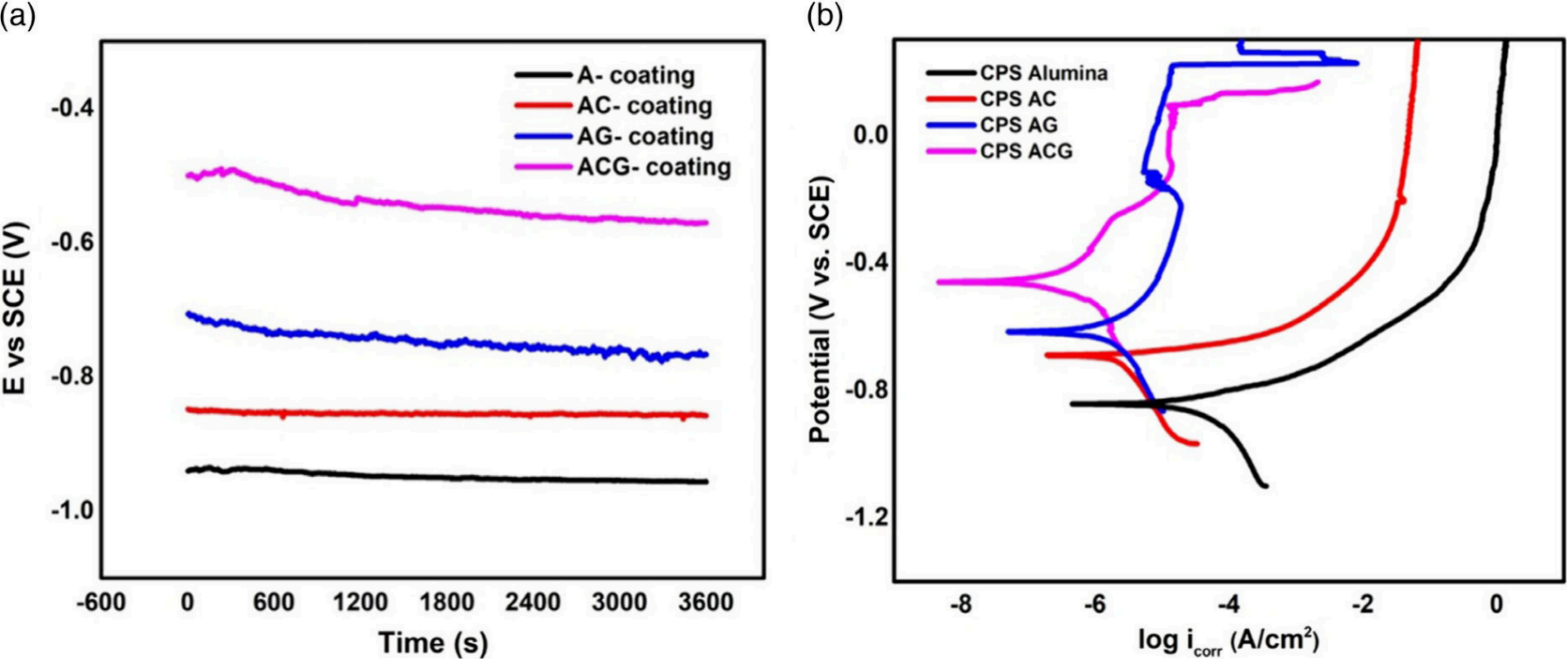
Corrosion performance of the coatings, as depicted by (a) open circuit potential (OCP); and (b) potentiodynamic polarization curve of the coatings.^26^ Figure taken/reproduced with permission from John Wiley and Sons, copyright [2019].

Beyond reducing porosity, CNTs and GNPs act as nanoscale barrier agents that obstruct ionic transport. GNPs, due to their two-dimensional structure and large aspect ratio, form impermeable barriers within the coating. Electrolytes attempting to penetrate the coating must travel around the nanosheets, creating a tortuous diffusion pathway that slows ingress. CNTs complement this mechanism by filling residual microvoids and bridging cracks, effectively sealing off preferential channels for electrolyte transport.^17,103–105^ Together, CNTs and GNPs create a hierarchical barrier system operating at multiple scales: nanosheets obstruct diffusion across splat lamellae, while nanotubes reinforce splat interfaces and fill voids. The electrochemical corrosion resistance of CNT/GNP reinforced Al_2_O_3_ coatings can further be attributed to a combination of structural and chemical mechanisms.^26^ At the structural level, reinforcement reduces interconnected porosity, strengthens splat interfaces and introduces tortuous diffusion paths that obstruct electrolyte ingress. At the chemical level, reinforcement stabilizes α-Al_2_O_3_, reducing the solubility and reactivity of the coating in aqueous environments. The synergy of these mechanisms explains why hybrid CNT/GNP coatings consistently outperform both monolithic Al_2_O_3_ and single-filler composites.

#### 4.2.5 Membrane Performance of CNT/GNP-Reinforced Al_2_O_3_ Nanocomposites

Ceramic membranes are increasingly recognized as attractive alternatives to polymeric membranes for wastewater treatment, desalination and industrial separations due to their superior thermal stability, chemical resistance and mechanical durability. Plasma spraying offers a scalable and cost-effective route for membrane fabrication, allowing for precise control over pore size and connectivity.^106–110^ The incorporation of CNTs and GNPs into plasma-sprayed Al_2_O_3_ membranes introduces a further dimension of tunability, significantly improving separation efficiency, fouling resistance and long-term stability, shown in Figure 11. As per our previous research, the first and most prominent benefit of CNT/GNP incorporation is enhanced rejection efficiency. Pure Al_2_O_3_ membranes typically exhibit dye or contaminant rejection in the range of 75–85% depending on pore structure. Our study demonstrated that hybrid CNT/GNP reinforced membranes achieved rejection efficiencies of over 98% for methylene blue and other model organic contaminants. This remarkable performance was attributed to two mechanisms: (i) densification of the microstructure, which reduced the size and continuity of pores, thereby filtering out smaller particles and (ii) additional adsorption sites provided by the high-surface-area carbon nanostructures.^86^ The results showing how the properties of the membranes improved after the nanofillers reinforcement have been improved, is shown in Figure 11. GNPs, with their planar morphology, offer abundant π–π interactions with dye molecules, while CNTs, with their tubular geometry, promote entrapment and surface binding. Together, these effects greatly enhance the selectivity of the membrane. Second major advantage is improved water flux and recovery after fouling cycles. In typical Al2O3 membranes, fouling—caused by deposition of organic matter or particulates on the membrane surface—leads to irreversible decreases in flux. Pure Al_2_O_3_ membranes often recover less than 85–90% of their initial flux after cleaning. In contrast, hybrid CNT/GNP reinforced membranes consistently recovered ∼97% of flux after repeated fouling–cleaning cycles. The improvement arises from the smooth and hydrophilic surfaces imparted by GNPs, which reduce the adhesion of foulants and the mechanical robustness conferred by CNTs, which prevents pore collapse during cleaning. As a result, contaminants are more easily removed during backwashing or chemical cleaning, restoring membrane performance.

**Figure 11:**
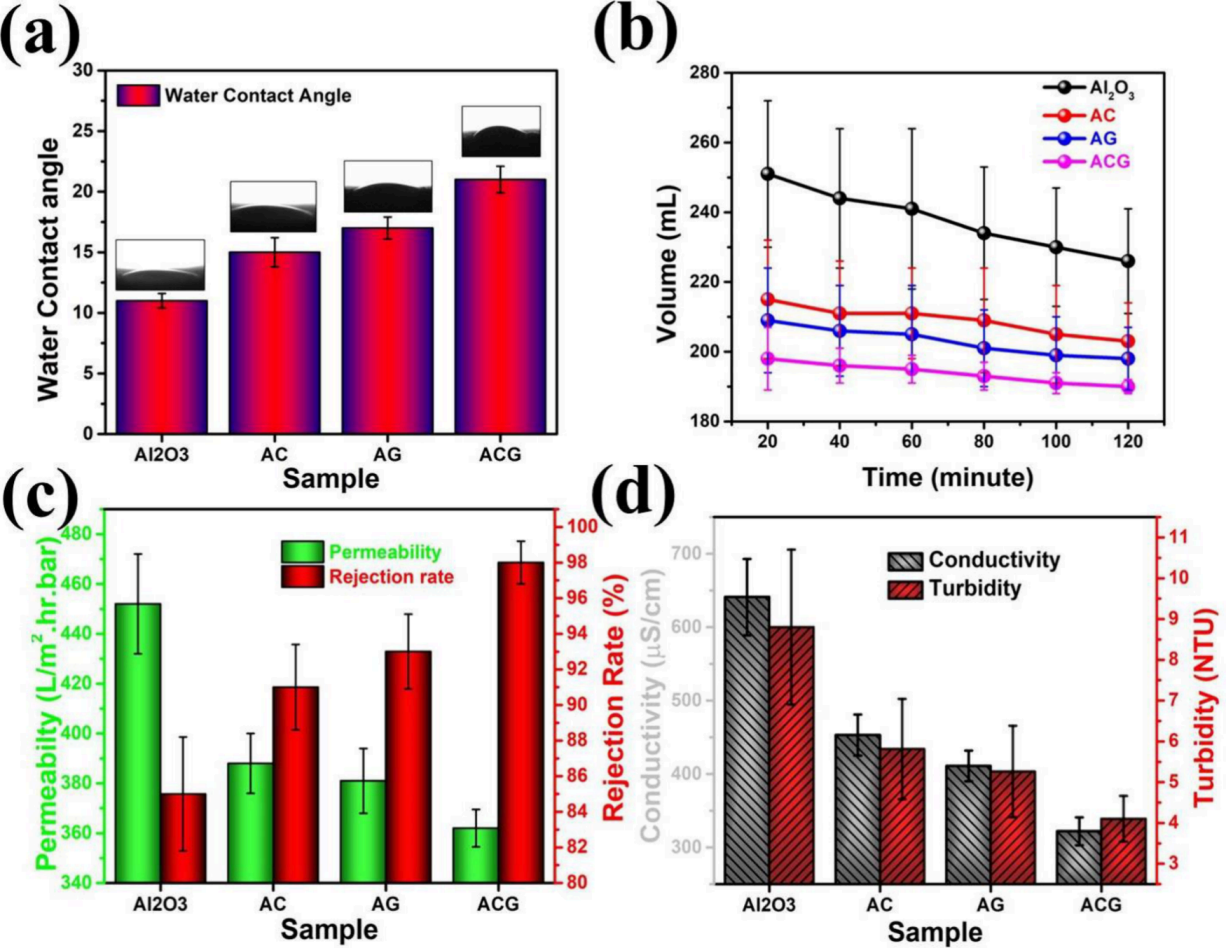
(a) Water contact angle of monolithic and reinforced coatings; (b) permeate volume collected with time; (c) effect of reinforcement on the permeability and rejection rate of the membranes; and (d) permeate characteristics for ensuring the quality after filtration.^86^ Figure taken/reproduced with permission from Elsevier, copyright [2019].

The presence of CNTs and GNPs also enhanced antifouling behavior during continuous operation. GNPs increase the hydrophilicity of the surface due to oxygen-containing functional groups that form during plasma spraying, thereby lowering the interfacial free energy between the membrane surface and water. This discourages hydrophobic foulant deposition, as shown in Figure 12.^86^ Additionally, CNTs embedded within the membrane backbone provide nanoscale reinforcement, resisting crack formation and ensuring that the pore structure remains stable under fluctuating pressures. Together, these effects minimize both reversible and irreversible fouling, thereby extending the operational life of the membrane. Another critical improvement due to CNT/GNP incorporation is mechanical durability under cyclic stresses. Conventional ceramic membranes are often brittle and prone to cracking under repeated pressurization or thermal cycling.^107,111–113^ CNTs act as nanoscale bridges across pores and splat boundaries, while GNPs distribute stresses along their basal planes, providing enhanced fracture resistance. Membrane study by Pandey et al. showed that hybrid nanocomposite membranes maintained their performance even after multiple cycles of backwashing and drying, conditions under which pure Al_2_O_3_ membranes often exhibited performance degradation due to microcracking.^86^ This durability ensures reliable operation in industrial settings, where membranes are subjected to harsh conditions.

**Figure 12:**
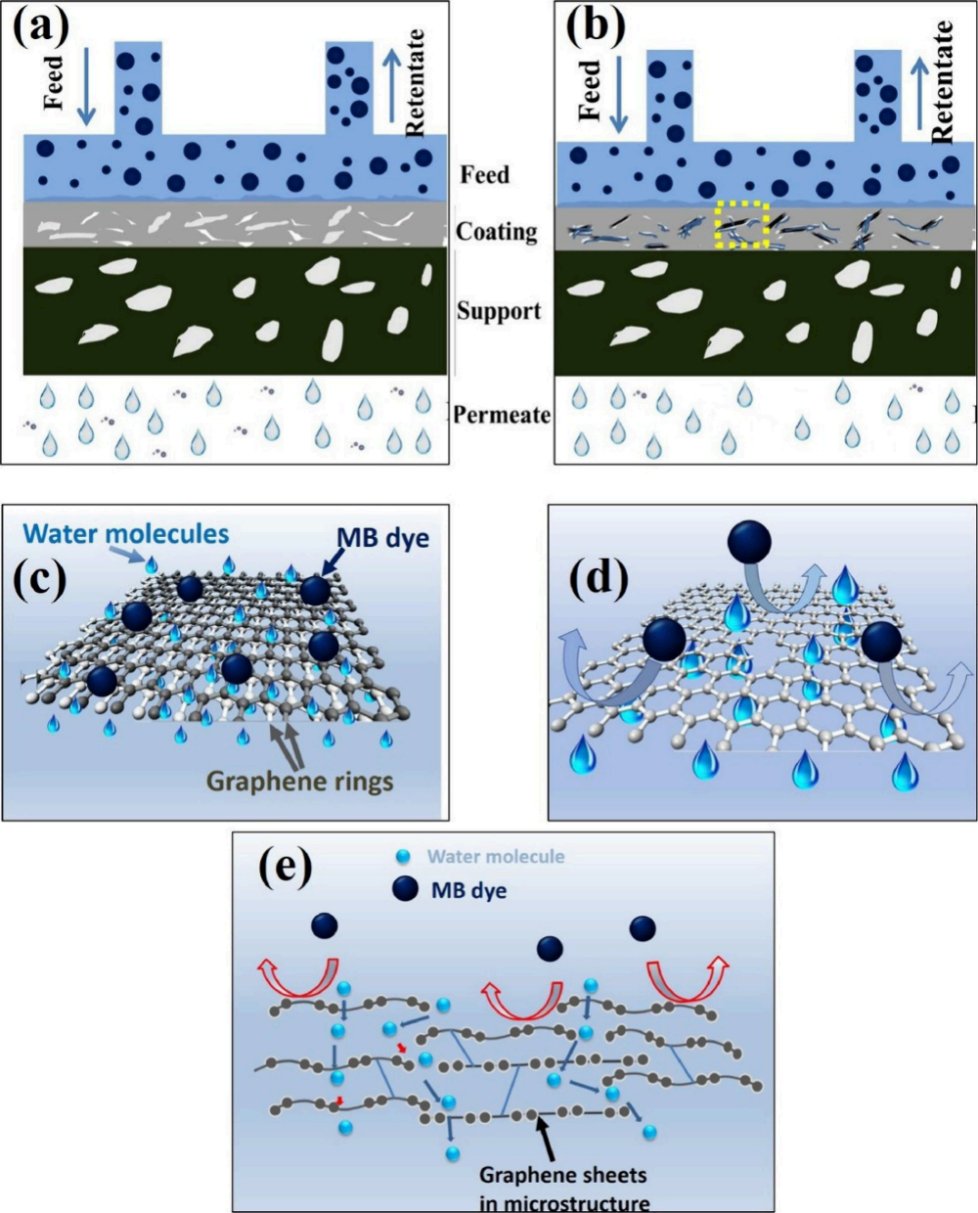
Schematic illustration showing the filtration mechanism through plasma-sprayed (a) Al_2_O_3_ and (b) ACG membranes. The incorporation of CNTs and GNPs enhances the filtration efficiency of Al_2_O_3_ membranes. (c) Enlarged region from (b) highlights possible filtration channels through CNT–GNP nanostructures, supported by (d) nanopores in graphene rings and (e) water transport between carbon layers.^86^ Figure taken/reproduced with permission from Elsevier, copyright [2019].

The performance enhancements extend beyond water filtration to broader environmental applications. The adsorption capacity introduced by CNTs and GNPs enables membranes to capture heavy metals, organic pollutants and even emerging contaminants such as pharmaceuticals. The combination of size-exclusion (due to refined pore structures) and adsorption (due to graphene sites) offers a dual-mode filtration mechanism that significantly improves overall membrane efficiency. For instance, GNPs can immobilize contaminants through electrostatic attraction or surface functional groups, while CNTs provide deep trapping within their tubular structures.

Complementary literature further supports these findings. Rahman et al. emphasized the role of hybrid nanofillers in reducing porosity and refining microstructure in SPS composites, effects that directly correlate with improved selectivity and durability in membrane systems.^97^ Similarly, another group observed that GNPs at grain boundaries provided structural stability and phase control, which in a membrane context translates to enhanced resistance to chemical attack.^34,38^ Taken together, these studies confirm that the improvements seen in plasma-sprayed hybrid membranes are not isolated but reflect fundamental reinforcement mechanisms. From a quantitative perspective, the improvements due to CNT and GNP reinforcement are striking. Dye rejection efficiencies increased from ∼ 80% in pure Al_2_O_3_ membranes to >98% in hybrids. Flux recovery after fouling rose from <90% to ∼ 97%. Membrane lifetimes under cyclic testing improved by at least 30–40% and fouling resistance indices showed reductions of 40–50% in irreversible fouling compared to conventional membranes. These data underscore the transformative impact of CNTs and GNPs on membrane performance.

## 5 Conclusions

The development of CNT- and GNP-reinforced Al_2_O_3_ nanocomposites represents a significant step toward overcoming the inherent limitations of conventional alumina, including porosity, weak intersplat bonding, and limited toughness and corrosion resistance. This review highlights that the improved performance of these systems is governed by well-defined reinforcement mechanisms. Individually, CNTs enhance toughness through crack bridging and load transfer, while GNPs promote splat spreading, lubrication, and diffusion barrier effects. When combined, their synergistic interaction enables multidirectional stress distribution, improved densification, and enhanced microstructural stability, resulting in superior overall performance. Quantitative comparisons across the literature reveal substantial improvements, including up to ∼ 60% increase in hardness, nearly 5-fold enhancement in adhesion strength, and significant gains in fracture toughness. In addition, electrochemical and membrane studies demonstrate marked improvements in corrosion resistance and filtration efficiency, confirming the multifunctional nature of these hybrid systems. A key contribution of this review is the mechanistic understanding of how hybrid nanocarbon reinforcements influence microstructure evolution and property enhancement at multiple scales. However, challenges related to uniform dispersion, thermal stability of reinforcements, and scalable processing remain. Future research should focus on advanced feedstock design, improved dispersion strategies, and process optimization to enable reliable large-scale application of these high-performance nanocomposites.

The integration of CNT/GNP hybrid reinforcements into plasma-sprayed alumina coatings offers transformative potential for several high-growth industrial sectors. In the field of heavy machinery and manufacturing, the enhanced fracture toughness and significantly reduced wear rates directly translate to an increased service life for pump seals, valve components, and bearing surfaces, potentially reducing maintenance downtime by an estimated 30–40%. Furthermore, the unique combination of chemical inertness and multiscale porosity control makes these composites ideal candidates for advanced filtration and corrosion-resistant membranes in harsh chemical processing environments. Beyond structural utility, the tailorable electrical conductivity of these hybrid coatings opens a niche market in electrostatic discharge (ESD) protection for aerospace components and smart sensors for real-time structural health monitoring. As plasma spraying is already a mature, high-throughput industrial process, the transition of these nanostructured coatings from the laboratory to the global coating market—valued at billions of dollars—is highly feasible, provided that cost-effective dispersion and large-scale feedstock consistency are maintained.

## 6 Future Outlook

While the synergistic potential of 1D-CNT/2D-GNP reinforced alumina coatings is evident, transitioning these materials from laboratory-scale studies to industrial applications requires addressing specific technical frontiers. To advance the field, the following research pathways are proposed:

### 6.1 Data-Driven Process Optimization via Machine Learning

The multivariable nature of plasma spraying—encompassing powder morphology, plasma enthalpy, and particle velocity—presents a complex optimization challenge. Future research should leverage Machine Learning (ML) frameworks, such as Gaussian process regression or neural networks, to establish predictive models for the structural integrity of carbon nanostructures. By correlates processing inputs with splat-level microstructural outputs, researchers can develop a “process-window map” that minimizes thermal degradation of the reinforcements while maximizing the interlamellar bonding of the alumina matrix.

### 6.2 Development of Architected and Hierarchical Interfaces

To move beyond the limitations of random reinforcement distribution, focus must shift toward in situ architectural design. Utilizing advanced delivery systems like Suspension Plasma Spraying (SPS) or Solution Precursor Plasma Spraying (SPPS) offers the potential to engineer “segregated″ networks. By strategically localizing CNTs and GNPs at the splat boundaries, a continuous three-dimensional skeleton can be formed. This hierarchical architecture is expected to significantly lower the electrical percolation threshold and provide superior crack-shielding capabilities through precisely controlled interfacial sliding.

### 6.3 Long-Term Reliability and Environmental Stability Assessments

For these composites to succeed in aerospace or energy sectors, their performance under cyclic extreme conditions must be quantified. There is a critical knowledge gap regarding the environmental aging and galvanic coupling between carbonaceous fillers and the ceramic matrix. Future studies should prioritize in situ high-temperature mechanical testing and thermal-cycling-coupled corrosion studies. Evaluating the interface stability over extended lifecycles will be essential for establishing the structural reliability required for real-world engineering components.
